# *COVID-19 Stats:* College and University* COVID-19 Student Testing Protocols,^†^ by Mode of Instruction^§^ (N = 1,849) — United States, Spring 2021^¶^

**DOI:** 10.15585/mmwr.mm7014a5

**Published:** 2021-04-09

**Figure:**
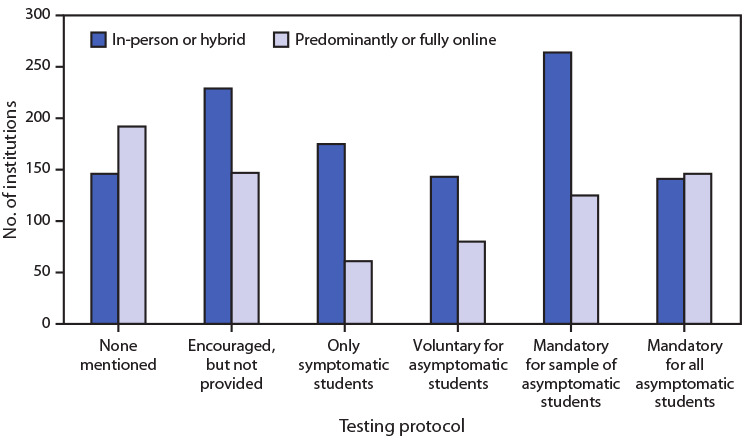
As of March 17, 2021, a total of 899 (49%) of 1,849 public and private, nonprofit 4-year U.S. colleges and universities provided some type of COVID-19 testing for asymptomatic students, including 548 (30%) institutions conducting classes in-person or in a hybrid format. Among institutions providing testing for asymptomatic students, 389 (43%) had protocols that required periodic testing for various subgroups (e.g., athletes, fraternity and sorority activity participants, and a random sample of students); 287 (32%) mandated that all students receive testing (ranging from every other day to once every other week), which did not vary by public or private, nonprofit status or by mode of instruction. Among institutions, 18% (338 of 1,849) did not mention a COVID-19 testing protocol on their websites, including146 with in-person or hybrid instruction. Although asymptomatic transmission is estimated to account for approximately one half of SARS-CoV-2 transmission, a majority (950; 51%) of institutions did not publish a testing protocol for screening asymptomatic students in spring 2021.

**Source:** Data from college and university websites collected by the College Crisis Initiative, Davidson College. https://www.collegecrisis.org

